# Eye movement changes as an indicator of mild cognitive impairment

**DOI:** 10.3389/fnins.2023.1171417

**Published:** 2023-06-15

**Authors:** Julius Opwonya, Boncho Ku, Kun Ho Lee, Joong Il Kim, Jaeuk U. Kim

**Affiliations:** ^1^Digital Health Research Division, Korea Institute of Oriental Medicine, Daejeon, South Korea; ^2^KM Convergence Science, University of Science and Technology, Daejeon, South Korea; ^3^Gwangju Alzheimer’s Disease and Related Dementias (GARD) Cohort Research Center, Chosun University, Gwangju, South Korea; ^4^Department of Biomedical Science, Chosun University, Gwangju, South Korea; ^5^Dementia Research Group, Korea Brain Research Institute, Daegu, South Korea

**Keywords:** Alzheimer’s disease, mild cognitive impairment, eye movement analysis and synthesis, machine learning (ML), saccades

## Abstract

**Background:**

Early identification of patients at risk of dementia, alongside timely medical intervention, can prevent disease progression. Despite their potential clinical utility, the application of diagnostic tools, such as neuropsychological assessments and neuroimaging biomarkers, is hindered by their high cost and time-consuming administration, rendering them impractical for widespread implementation in the general population. We aimed to develop non-invasive and cost-effective classification models for predicting mild cognitive impairment (MCI) using eye movement (EM) data.

**Methods:**

We collected eye-tracking (ET) data from 594 subjects, 428 cognitively normal controls, and 166 patients with MCI while they performed prosaccade/antisaccade and go/no-go tasks. Logistic regression (LR) was used to calculate the EM metrics’ odds ratios (ORs). We then used machine learning models to construct classification models using EM metrics, demographic characteristics, and brief cognitive screening test scores. Model performance was evaluated based on the area under the receiver operating characteristic curve (AUROC).

**Results:**

LR models revealed that several EM metrics are significantly associated with increased odds of MCI, with odds ratios ranging from 1.213 to 1.621. The AUROC scores for models utilizing demographic information and either EM metrics or MMSE were 0.752 and 0.767, respectively. Combining all features, including demographic, MMSE, and EM, notably resulted in the best-performing model, which achieved an AUROC of 0.840.

**Conclusion:**

Changes in EM metrics linked with MCI are associated with attentional and executive function deficits. EM metrics combined with demographics and cognitive test scores enhance MCI prediction, making it a non-invasive, cost-effective method to identify early stages of cognitive decline.

## 1. Introduction

Alzheimer’s disease (AD) is a progressive neurodegenerative disorder characterized by the accumulation of amyloid beta (Aβ) plaques and neurofibrillary tau-based tangles, beginning decades before symptoms appear and lead to cognitive decline, with individuals progressing from normal cognitive abilities to prodromal AD and ultimately AD dementia (Jack et al., 2018). While treatment can ameliorate some symptoms of dementia, there is no currently available cure, and the disease inevitably progresses (Alzheimer’s Association, 2022). Early diagnosis and intervention during the mild cognitive impairment (MCI) stage are essential to preventing the progression to dementia and improving the quality of life for those with preclinical or prodromal AD (Gauthier et al., 2006).

Validated biomarkers that are proxies for AD neuropathologic changes exist but are underutilized due to their invasive, high cost, and limited availability (Jack et al., 2018). A neuropsychological evaluation is the most widespread method used in clinical settings to screen for cognitive impairment and obtain a global index of cognitive functioning (Nasreddine et al., 2005; McKhann et al., 2011; Bradfield, 2021). Evaluations range from simple bedside tests and brief screening tools to detailed neuropsychological batteries. Brief cognitive screening tests have been used and refined throughout the years, including the Mini-Mental State Examination (MMSE), Mini Cognitive Assessment Instrument (Mini-Cog), and Montreal Cognitive Assessment (MoCA) (Bradfield, 2021). Brief cognitive measures, such as the MMSE that can be easily administered with minimal training are optimal for fast-paced, high-patient-volume screening settings that often encounter older adult patients with cognitive problems. Although an array of brief cognitive screening tools that are sensitive to AD exist, most depend on intact linguistic function, which is highly influenced by demographic variables, and they may not be sensitive to the early stages of cognitive impairment (Nasreddine et al., 2005).

Furthermore, several cognitive tests entail writing and drawing, and motor dysfunction is highly prevalent in dementia patients, which can impact the results. Therefore, the utility of these brief cognitive tests, especially in patients with late-stage AD, can be limited. The gold standard for cognitive examination is a neuropsychological assessment battery, which requires in-depth training to ensure standardized administration and accurate interpretation of the findings (McKhann et al., 2011). Neuropsychological battery testing is not typically feasible in fast-paced clinical settings, such as primary care facilities. A rapid and easy-to-administer, non-invasive screening tool that is accurate and sensitive to the detection of MCI and AD dementia could accelerate new therapeutics for AD by selecting good trial candidates in the preclinical or prodromal stages and also screen healthy individuals in primary care facilities (Cummings et al., 2021).

Machine learning models built using non-invasive patient data are obvious candidates for use as screening tools. Previous research has demonstrated the use of machine learning in classifying MCI/AD dementia patients and cognitively normal controls and the potential of speech and language-based tools for non-invasive AD risk stratification (de la Fuente Garcia et al., 2020; Pulido et al., 2020; Tanveer et al., 2020). A major challenge in implementing a large-scale language tool is the presence of linguistic differences among speakers of different languages and dialects, which can result in variations in expression, speaking speed, and word usage. For these reasons, there are gaps between the clinical potential, research contexts, and actual clinical implementations of these tools.

Another modality gaining momentum is eye movement (EM) analysis; in recent years, eye-tracking (ET) devices have provided adequate temporal resolution, accuracy, and precision for measuring EM and detecting changes in pupil diameter (Klingner et al., 2008; Tobii Technology AB, 2012). The ET technique provides a non-invasive, quantitative, and objective evaluation of EM, which researchers can apply to assess cognitive function. Recent research suggests that impaired EM may be an early indicator of AD (Anderson and MacAskill, 2013), evident even in the prodromal stage and worsening as the disease progresses (Albers et al., 2015; Kusne et al., 2017); EM metrics may be used as biomarkers of both disease status and progression. Promising findings have emerged recently on the predictive value of EM data collected during reading tasks alone or combined with language data during reading activities (Biondi et al., 2018; de la Fuente Garcia et al., 2020).

The present study aimed to investigate the potential utility of EM data collected during an interleaved paradigm in differentiating individuals with MCI from CN controls. Specifically, we aimed to investigate the potential of EM data, either independently or in combination with neuropsychological scores, to improve the accuracy of distinguishing between individuals with MCI and CN controls. We collected demographic information, cognitive scores, and EM metrics from participants who completed the PS/AS and Go/No-go tasks. We explored the potential benefits of combining these datasets with MMSE scores. This study sheds light on the potential of EM data as a novel biomarker for MCI and examines the advantages of using a multimodal approach for improving prediction accuracy.

## 2. Methods

### 2.1. Participants

In total, 679 individuals participated in the study between October 2019 and December 2020. We divided the participants into MCI patient and age-matched CN control groups. MCI patients and CN controls were recruited at the Gwangju Alzheimer’s Disease and Related Dementia (GARD) center (Gwangju City, South Korea) (Doan et al., 2022).

We examined all the participants through detailed clinical consultations, incorporating a neuropsychological battery and the Clinical Dementia Rating (CDR) scale. CN controls were identified clinically as those who had a CDR score of zero and no sign of cognitive impairment; those with a CDR score of 0.5 and evidence of cognitive decline in one or more domains were considered MCI (Albert et al., 2011). MCI patients had a Seoul Neuropsychological Screening Battery-Second Edition (SNSB-II) *z score* of less than −1.5 in at least one of the domains. The SNSB-II is a widely used tool in South Korea for evaluating cognitive function in patients with MCI and dementia (Kang et al., 2003). In our study, all participants were assessed using the Korean version of the MMSE, which was included in the SNSB-II (Park, 1989).

Potential study participants underwent magnetic resonance imaging (MRI) scans to screen for evidence of brain atrophy or other focal brain lesions. Exclusion criteria for the study were as follows: the presence of focal brain lesions on MRI, including lacunes and white matter hyperintensity lesions of grade 2 or more (Fazekas et al., 1987; Kim et al., 2008); less than 3 years of education; and medical conditions that could interfere with the study design, such as mental health instability or a history of excessive alcohol consumption. A total of 85 participants were excluded based on these criteria. Specifically, we excluded 25 individuals diagnosed with AD dementia, 60 participants with visual impairments, and those who failed calibration and the preliminary trials that were conducted to familiarize participants with the task requirements before the actual trial. A total of 594 participants were included in the final analysis, including 428 CN controls and 166 MCI patients, as shown in Table 1. The CN group included 428 subjects (190 males, 238 females), with a mean age of 71.2 ± 6.2 years; the MCI group included 166 subjects (83 males, 83 females), with a mean age of 73.5 ± 6.6 years (see Table 1). We obtained written informed consent from all participants or their legal guardians after providing a detailed description of the study, which was approved by the Chonnam National University Hospital Institutional Review Board (IRB no CNUH-2019-279).

**Table 1:** Participants’ demographic information and neuropsychological test scores.

Characteristic	All^1^ (*N* = 594)	MCI^1^ (*N* = 166)	CN^1^ (*N* = 428)	*p* value^2^
Sex (female)	321 (54%)	83 (50%)	238 (56%)	0.2
Age	71.80 (6.42)	73.45 (6.63)	71.17 (6.22)	**<0.001**
Education level	13.0 (4.4)	12.9 (4.5)	13.1 (4.4)	0.8
MMSE score	27.32 (2.16)	26.16 (2.64)	27.77 (1.75)	**<0.001**

1 The values represent the mean (SD) for continuous variables and *n* (%) for categorical variables. The *p* values for the continuous variables were obtained using the Wilcoxon rank sum test. For the categorical variables, the *p* values were derived from the Chi-squared test statistics.

2 Pearson’s Chi-squared test; Wilcoxon rank sum test. The bold fonts indicate a *p* value lower than 0.05.

### 2.2. Eye-tracking recordings and the experimental paradigm

The standardized pipeline used to preprocess the gaze data collected for each participant has been described in detail elsewhere (Opwonya et al., 2022). We recorded SEM data on the Tobii Pro spectrum system (Tobii Pro AB, Danderyd, Sweden), sampled at 300 Hz, and processed with Tobii Pro Lab version 1.118. Visual stimuli were presented on a monitor approximately 65 centimeters from the participants. Furthermore, we used a desk with adjustable chin and forehead rests to maintain a suitable angle between each participant’s gaze and the ET monitor.

**Figure 1:**
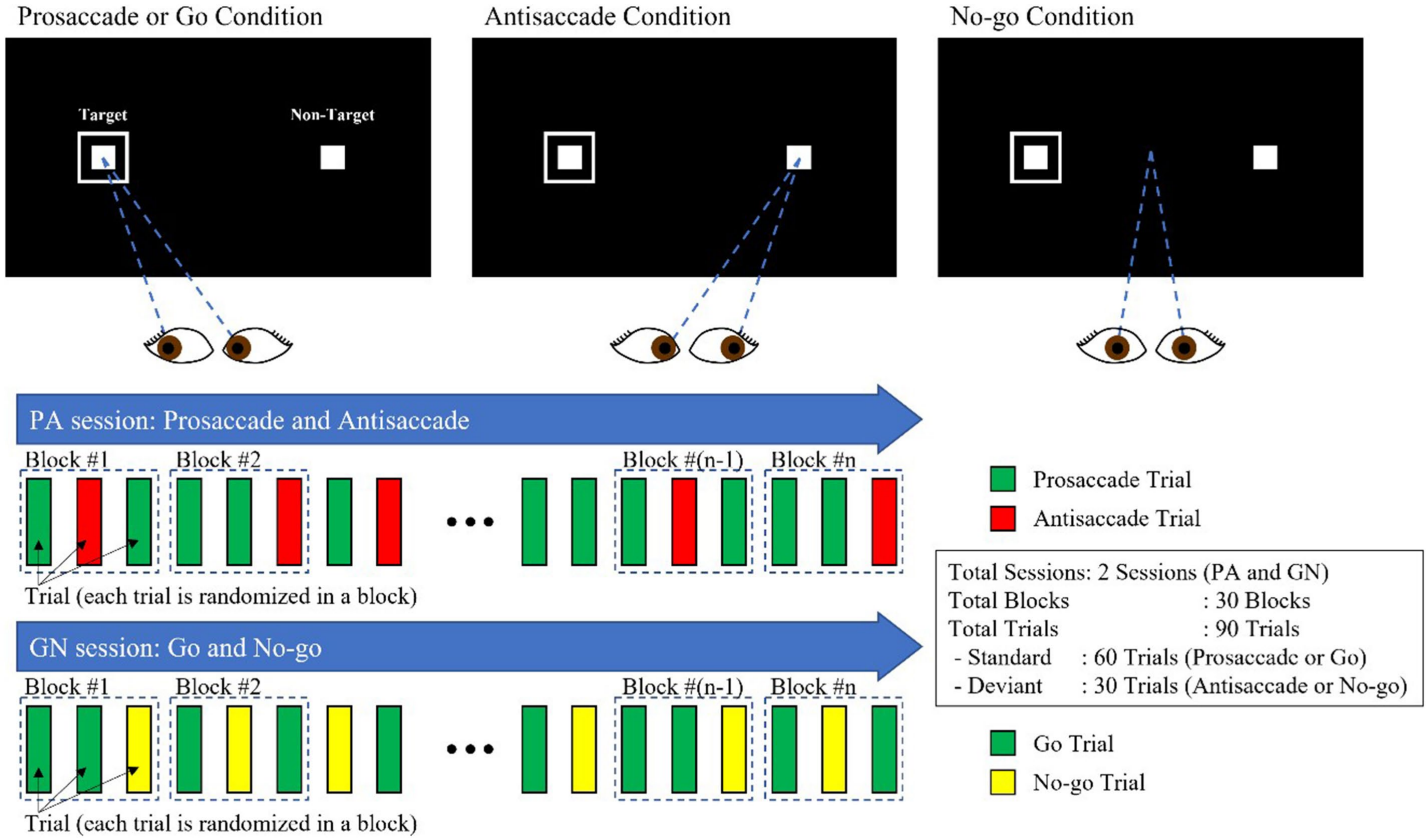
PS/AS and Go/No-go paradigms.

Our experimental design involved two interleaved sessions: the PS/AS (PA) and the Go/No-go (GN). Each session consisted of 30 blocks, each consisting of 3 trials, with two standard (PS and Go) trials and one deviant trial (AS and No-go), as shown in Figure 1. The condition (PS, AS, Go, and No-go) and the peripheral target, projected at ±10° in the horizontal plane (left/right), were randomly interleaved with an equal frequency throughout each block.

This study classified six EM-related responses, including fixation duration, correct responses, anticipations, omissions, corrected inhibition errors, and uncorrected inhibition errors. We classified all errors as the summation of anticipatory errors, omissions, and inhibition errors in the PS/AS and Go trials and all errors as the summation of inhibition errors in the No-go trials. Criteria were established to determine the PS/AS and Go/No-go responses.

Correct responses for PS and Go trials were defined as the first saccade directed toward the target location, followed by a sustained fixation within the area of interest (AOI). For AS trials, correct responses were defined as the first saccade in the opposite direction of the target location, followed by sustained fixation within the AOI. In No-go trials, correct responses were identified as a maintained fixation at the center of the screen despite the appearance of any directional targets, indicating successful inhibition.

Anticipatory errors were defined as the first saccade that occurred less than 80 ms following target stimulus onset, while omission errors were defined as the absence of eye movement within 500 ms of target presentation. Inhibition errors were further subcategorized into corrected and uncorrected errors. Uncorrected inhibition errors were trials where the target stimuli inappropriately captured the gaze, but no corrective saccade was made. Corrected inhibition errors were defined as errors in which the gaze was redirected from an incorrect to a correct direction within 400 ms, with a gaze variation ≤1°.

### 2.3. Statistical analysis

R version 4.2.2, including the *gtsummary* (v.1.6.2) and *tidyverse* packages (v.1.3.2), was used for statistical analysis (Wickham, 2017; Sjoberg et al., 2021; R Core Team, 2022). We performed binomial logistic regression analyses to examine the ability of EM metrics to distinguish between CN controls and MCI patients. We estimated ORs from two logistic regression models (crude and adjusted), which included adjustments for age, sex, and education level.

### 2.4. Machine learning and model selection

We used the *mikropml* package (v.1.4.0) to train and evaluate models to predict cognitive status from the EM metrics obtained from PS/AS and Go/No-go tasks. Demographics, cognitive scores, and ET metric data were used to generate logistic regression (LR), random forest (RF), support vector machine (SVM), and extreme gradient boosting (XGB) classification models to predict cognitive status.

We aimed to classify patients with MCI (vs. CN controls) using each feature set (see below); we built classifiers using demographic characteristics, MMSE scores, and EM metric data. We performed LR on all feature sets using a modified version of the machine learning pipeline presented in the study by Topcuoglu et al. (2021) and caret version 6.0-93 in R version 4.2.2. Furthermore, we performed RF, SVM with a radial basis kernel, and XGB classification for the feature sets using the same method implemented in *mikropml* (Topcuoglu et al., 2021). We randomly split the data into 80/20 train/test splits, and the train/test splits were identical across models generated with different feature sets for a valid comparison.

Given that the data were imbalanced, we applied the synthetic minority oversampling technique (SMOTE) during cross-validation, which allowed for proper evaluation of the model’s capability to generalize from the training data and avoided biases or overly optimistic estimates (Santos et al., 2018). Hyperparameters were selected via cross-validation on the training set to maximize the average area under the receiver operating characteristics curve (AUROC) across cross-validation folds.

### 2.5. Feature sets

We studied the relationship between cognitive status and six feature sets: (i) demographic characteristics—sex, age, and years of education; (ii) MMSE scores; (iii) demographic characteristics and MMSE scores; (iv) EM features—variables derived from the PS/AS and Go/No-go tasks; (v) demographic characteristics and EM features; and (vi) demographic characteristics, MMSE scores, and EM features. Demographic features (particularly age, sex, and years of education) can be obtained noninvasively and are predictive of dementia in previous studies (Calvin et al., 2019).

### 2.6. Feature set preprocessing

We preprocessed all six datasets by mapping categorical features to binary variables, centering and scaling the continuous features, and removing features present in only one sample or all but one sample.

### 2.7. Baseline classifier

We selected the best available classification method using demographic information (age, sex, and education level) as the baseline for our algorithm development (see Table 1).

### 2.8. Model performance

To characterize the accuracy of the MMSE scores and EM metrics, we performed receiver operating characteristic (ROC) curve analyses to calculate the AUROC and the deviance across all train/test splits. We selected the optimal model based on the highest AUROC and lowest binomial deviance in each combination of learning algorithms and six feature sets. Subsequently, we evaluated the predictive performance of this model on the test set. Feature importance was calculated using a permutation test, which breaks the relationship between the feature and the true outcome in the test data and measures the change in model performance.

## 3. Results

### 3.1. Participant characteristics

The participants’ baseline demographic characteristics and cognitive scores are shown in Table 1. Patients with MCI were significantly older (*p* < 0.001) and had significantly lower MMSE total scores (*p* < 0.001) than CN controls. There was no difference in sex or years of education between the groups.

### 3.2. Eye-movement tasks

Several EM variables were significantly predictive of MCI on all tasks, even after being adjusted for demographics.

### 3.3. Prosaccade tasks

Univariate logistic regression models adjusted for demographics showed increased odds of MCI for individuals with wider latency variability (OR 1.532, 95% CI 1.262–1.868, *p* < 0.001), more errors (OR 1.348, 95% CI 1.106–1.645, *p* = 0.003), and more anticipations (OR 1.213, 95% CI 1.006–1.461, *p* = 0.043) (see Table 2).

**Table 2:** Estimated odds ratios and 95% confidence intervals of Pro/antisaccade EM variables derived from the two logistic regression models.

	Crude model			Adjusted model		
Variables	OR^1^	95% CI^2^	*p* value	OR^1^	95% CI^2^	*p* value^3^
**Prosaccade**						
Correct	0.717	0.597, 0.858	**<0.001**	0.736	0.601, 0.899	**0.003**
Latency	1.171	0.98, 1.400	0.082	1.137	0.945, 1.367	0.173
Latency SD	1.564	1.305, 1.883	**<0.001**	1.532	1.262, 1.868	**<0.001**
All errors	1.399	1.170, 1.677	**<0.001**	1.348	1.106, 1.645	**0.003**
Uncorrected error	1.115	0.936, 1.319	0.219	1.082	0.904, 1.286	0.381
Self-corrected	1.017	0.848, 1.214	0.857	0.998	0.829, 1.197	0.986
Anticipations	1.245	1.047, 1.480	**0.014**	1.213	1.006, 1.461	**0.043**
Omissions	1.195	1.008, 1.414	**0.041**	1.137	0.95, 1.353	0.151
**Antisaccade**						
Correct	0.614	0.501, 0.745	**<0.001**	0.621	0.495, 0.771	**<0.001**
Latency	0.878	0.732, 1.051	0.158	0.867	0.719, 1.043	0.131
Latency SD	0.97	0.807, 1.158	0.725	0.929	0.774, 1.112	0.426
All errors	1.642	1.353, 2.009	**<0.001**	1.604	1.295, 2.002	**<0.001**
Uncorrected error	1.447	1.221, 1.722	**<0.001**	1.394	1.171, 1.665	**<0.001**
Corrected error	0.702	0.574, 0.852	**<0.001**	0.713	0.581, 0.868	**<0.001**
Anticipations	1.297	1.088, 1.545	**0.004**	1.257	1.041, 1.517	**0.018**
Omissions	1.224	1.033, 1.448	**0.020**	1.178	0.99, 1.400	0.066

1 OR, odds ratio.

2 CI, confidence interval; SD, standard deviation.

3 *p* value obtained from the Wald test. The models were adjusted for age, sex, and years of education. The bold font indicates a *p* value lower than 0.05.

### 3.4. Antisaccade tasks

Univariate logistic regression models adjusted for demographics revealed increased odds of MCI for individuals with more errors (OR 1.604; 95% CI 1.295–2.002; *p* < 0.001), more uncorrected errors (OR 1.394; 95% CI 1.171–1.665; *p* < 0.001), and more anticipations (OR 1.041; 95% CI 1.041–1.517; *p* = 0.018) (see Table 2).

### 3.5. Go tasks

Univariate logistic regression models adjusted for demographics showed increased odds of MCI for individuals with a wider latency variability (OR, 1.216; 95% CI, 1.003–1.477; *p* = 0.047), more errors (OR, 1.555; 95% CI 1.274–1.906; *p* < 0.001), more anticipations (OR, 1.307; 95% CI 1.086–1.554; *p* = 0.0053), and more omissions (OR, 1.295; 95% CI 1.084–1.554; *p* = 0.0053) (see Table 3).

**Table 3:** Estimated odds ratios and 95% confidence intervals of Go/No-go EM variables derived from the two logistic regression models.

	Crude model			Adjusted model		
Variables	OR^1^	95% CI^2^	*p* value	OR^1^	95% CI^2^	*p* value^3^
**Go**						
Correct	0.610	0.507, 0.732	**<0.001**	0.616	0.501, 0.753	**<0.001**
Latency	1.227	1.028, 1.467	**0.024**	1.174	0.98, 1.413	0.090
Latency SD	1.269	1.061, 1.522	**0.009**	1.216	1.003, 1.477	**0.047**
All errors	1.596	1.332, 1.919	**<0.001**	1.555	1.274, 1.906	**<0.001**
Uncorrected error	1.177	0.99, 1.393	0.061	1.141	0.96, 1.355	0.135
Self-corrected	0.948	0.788, 1.134	0.564	0.935	0.775, 1.122	0.473
Anticipations	1.334	1.123, 1.585	**0.001**	1.307	1.086, 1.572	**0.005**
Omissions	1.365	1.152, 1.626	**<0.001**	1.295	1.084, 1.554	**0.004**
**No-go**						
Correct	0.604	0.505, 0.721	**<0.001**	0.604	0.497, 0.731	**<0.001**
Fixation duration	0.692	0.581, 0.822	**<0.001**	0.712	0.592, 0.854	**<0.001**
Fixation duration SD	1.503	1.240, 1.837	**<0.001**	1.458	1.198, 1.788	**<0.001**
All errors	1.603	1.346, 1.912	**<0.001**	1.598	1.324, 1.933	**<0.001**
Uncorrected error	1.621	1.362, 1.935	**<0.001**	1.621	1.343, 1.961	**<0.001**
Corrected error	0.996	0.823, 1.184	0.964	0.949	0.779, 1.135	0.579

1 OR, odds ratio.

2 CI, confidence interval; SD, standard deviation.

3 *p* value obtained from the Wald test. The models were adjusted for age, sex, and years of education. The bold font indicates a *p* value lower than 0.05.

### 3.6. No-go tasks

Univariate logistic regression models adjusted for demographics showed increased odds of MCI for individuals with a wider fixation variability (OR, 1.454; 95% CI, 1.198–1.788; *p* < 0.001), more errors (OR, 1.598; 95% CI 1.324–1.933; *p* < 0.001), and more uncorrected errors (OR, 1.621; 95% CI 1.343–1.961; *p* < 0.001) (see Table 3).

### 3.7. Diagnostic performance of the feature sets

#### 3.7.1. Baseline demographic characteristics

Table 4 shows the performance of the four classification algorithms (LR, RF, SVM, and XGB) and summarizes the diagnostic performance of the six feature sets. The LR algorithm exhibited the highest AUROC of 0.656 with deviance of 161.409; hence, we used it as the baseline for all subsequent experiments.

**Table 4:** AUROC results of prediction models according to feature set and classification model.

	Logistic regression		Random forest		Support vector machine		Extreme gradient boosting	
	AUROC	Deviance	AUROC	Deviance	AUROC	Deviance	AUROC	Deviance
Demo	**0.656**	161.409	0.552	–	0.610	–	0.613	158.382
MMSE	0.742	161.418	0.593	–	**0.743**	–	0.671	161.908
EM	0.702	149.244	**0.715**	–	0.643	–	0.707	148.732
Demo + MMSE	**0.767**	135.817	0.610	–	0.764	–	0.717	139.122
Demo + EM	0.700	149.333	**0.752**	–	0.718	–	0.726	137.378
Demo + MMSE + EM	0.773	138.866	0.831	–	0.769	–	**0.840**	121.671

Values in bold in each column represent the highest performance.

#### 3.7.2. Classification performance

**Figure 2:**
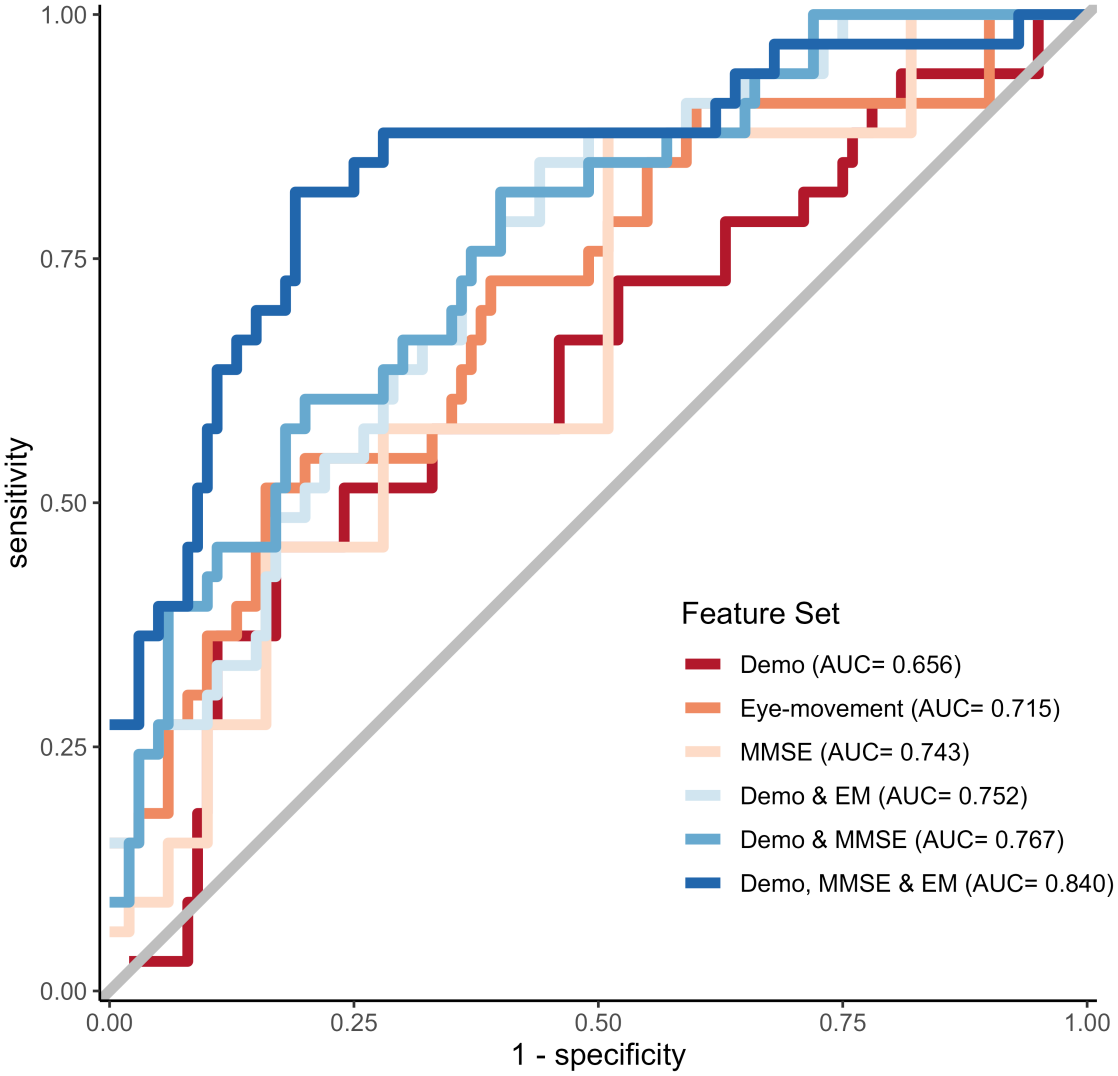
ROC curves for the best-performing prediction models per feature set.

In this study, we compared different models to determine the best predictors of cognitive impairment. Specifically, we examined the performance of a baseline model against two other models: one that included demographics and EM and another that included demographics and MMSE total score. The model incorporating EM as a predictor showed superior performance with an AUROC of 0.715, surpassing the baseline model that used only demographic information. However, the MMSE total score had a slightly higher AUROC than the EM features alone with AUROC of 0.743. Furthermore, we observed that adding demographics to either the EM or MMSE features independently resulted in further performance improvements, with AUROCs of 0.752 and 0.767, respectively. Finally, we evaluated the performance of a combined model that included all features, namely demographics, MMSE total score, and EM. Our analysis demonstrated that this model had the best overall performance, with an AUROC of 0.840 and lowest deviance of 121.671, as shown in Figure 2 and Table 4. Incorporating EM into predictive models may improve their accuracy in identifying cognitive impairment, and a combination of demographics, MMSE total score, and EM can effectively predict cognitive impairment, suggesting the importance of using multiple features in clinical assessments. Additional performance metrics, such as sensitivity and specificity, are provided in Supplementary Appendix Table A1.

**Figure 3:**
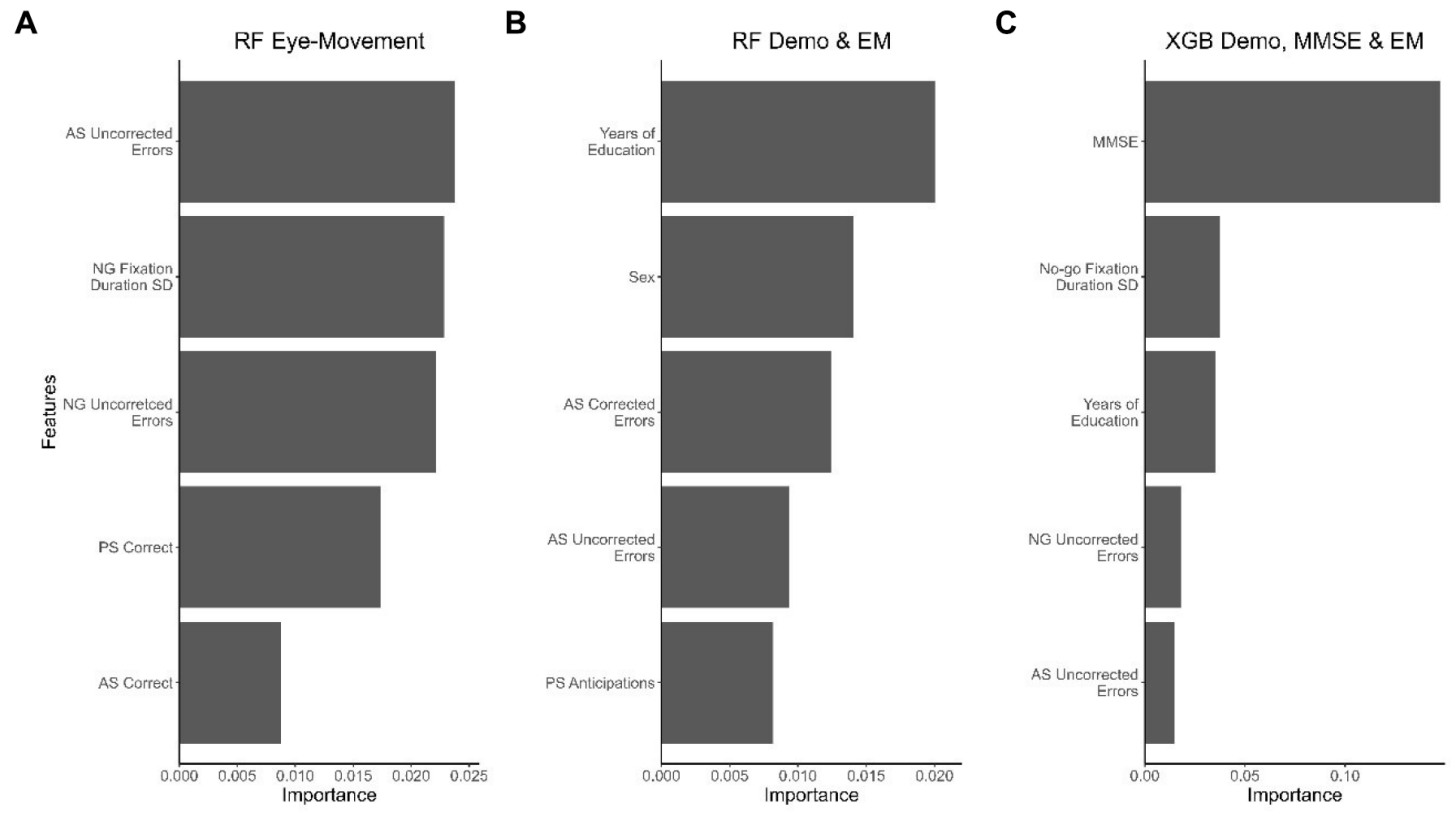
The top 5 features that contributed the most to the best-performing model in each dataset. **(A)** RF model with the EM dataset, **(B)** RF model with demographic and EM data, and **(C)** XGB model with demographic data, MMSE scores, and EM data. Important features for the LR and SVM models were selected according to decreasing importance to the AUROC value when the feature was permuted.

To determine each feature set’s contribution to the classification model’s performance, we calculated the feature importance using a permutation test, identifying the five most important features, as shown in Figure 3.

## 4. Discussion

This study evaluated the efficacy of EM metrics for screening MCI patients from CN controls, comparing these metrics individually or jointly with other easily accessible and cost-effective measures such as demographic information and MMSE scores. Specifically, we developed and validated prediction models with the ability to identify individuals with MCI using an ET paradigm comprising PS/AS and Go/No-go tasks and examining the contributions of demographic information, MMSE scores, and EM data.

The cohort EM variables that were consistently associated with MCI were latency variability, fixation duration variability, the number of errors, anticipations, and omissions (Opwonya et al., 2022). Kapoula et al. (2010) suggested that a wide latency distribution is a good index of attentional fluctuation when participants perform SEM tasks. Therefore, the greater latency variability in the MCI group suggests more extended moments of inattention where their focus drifted from the task to other irrelevant things. Fixation duration, which represents the relative focus on an object, with a greater average fixation time indicating a greater degree of attention (Tullis and Albert, 2013), was shorter and had more variability in the MCI group, indicating a disengagement of attention. Participants with difficulties in sustained attention or working memory, such as the MCI group in our study, showed increased odds of omissions in Go and AS tasks, further supporting the suggestion that they have attention deficiencies (Crawford et al., 2013). Our results suggested that the MCI group had poorer sustained attention than the CN group, as indicated by the greater saccade latency variability, shorter fixation duration, and more frequent omissions.

When a participant preempts the onset signal of a task with an anticipatory EM in advance of the target, anticipation errors are generated (Crawford et al., 2013). The MCI group showed increased odds of making anticipatory saccades. The higher frequency of anticipatory errors in the MCI group sheds light on the inhibitory dysfunction in these patients. Furthermore, we evaluated corrected and uncorrected inhibition errors to assess specific inhibitory processes. We found that the MCI group had increased odds of not correcting errors, consistent with an impairment in error monitoring. These findings are consistent with previous results, which showed that PS/AS and Go/No-go tasks could demonstrate specific deficits in inhibitory control, latency, and self-monitoring in patients with MCI compared to CN controls (Opwonya et al., 2022). Previous studies have linked variables such as latency variability, fixation duration variability, errors, anticipations, and omissions with increased odds of MCI or AD dementia (Crawford et al., 2013; Noiret et al., 2018; Opwonya et al., 2021, 2022) and likely contribute to cognitive function deficits in this patient group.

Next, we employed machine learning models to evaluate the predictive performance of EM features from the PS/AS and Go/No-go tasks, demographic information, and MMSE scores in a cohort of individuals with and without cognitive impairment. Upon comparing the EM feature performance to the baseline model, we found the EM metrics reliable for distinguishing MCI patients from CN. The results showed that the models using EM features alone outperformed those using demographic characteristics alone. Specifically, the discriminative ability of the ET model was superior in this dataset, with a peak AUROC of 0.715, compared to the demographic characteristics model, with an AUROC of 0.656. However, the MMSE alone had a higher performance with an AUROC of 0.743 than EM features. Incorporating demographic characteristics, MMSE scores, and EM data in the prediction model further improved the detection of MCI, yielding the highest AUROC of 0.840 and lowest deviance of 121.671. These findings suggest that EM metrics captured during PS/AS and Go/No-go tasks can help detect subtle cognitive impairment and have additive clinical utility when combined with demographic characteristics and MMSE scores.

Previous research has shown that the MMSE and Montreal cognitive assessment (MoCA) are effective diagnostic tools for dementia (Roalf et al., 2013). The MoCA is more sensitive and accurate in differentiating MCI patients from CN individuals than the MMSE (Nasreddine et al., 2005; Roalf et al., 2013). Longitudinal assessments are essential for determining the progression from MCI to AD dementia or other forms of dementia in clinical and research settings. Additionally, these assessments assist in differential diagnosis and the measurement of the treatment effectiveness. However, conducting serial assessments can be challenging due to practice effects, particularly when using brief cognitive tests like MMSE and MoCA. Utilizing eye-tracking technology can reduce potential confounding associated with repeated task exposure, although it does not eliminate carryover effects from the test–retest method. Compared to high-cost or invasive techniques, non-invasive tools such as eye trackers provide data that may not require expert interpretation in the clinical context and pose no risk to patients. The ability of non-experts to interpret EM metrics will rely on establishing consistent performance by simple or automated classification algorithms, irrespective of potential confounding factors such as age and educational level. Dementia pathology causes progressive neurodegeneration, resulting in altered oculomotor performance and a decline in cognitive functions (Molitor et al., 2015; Barral et al., 2020). Previous studies have demonstrated a considerable impact of aging on saccadic reaction times, as younger adults exhibit notably quicker mean reaction times than their older counterparts (Polden et al., 2020). Our study revealed weak to moderate correlations between demographics, specifically age and level of education, and EM variables. The correlation coefficients for these relationships are provided in Supplementary Appendix Figures A1, A2.

Furthermore, aging effects have been observed in oculomotor function, specifically in processing speed, spatial memory, and inhibitory control (Salthouse, 1996; Peltsch et al., 2011; Crawford et al., 2017). Eye movement changes in psychiatric disorders have been extensively studied, with schizophrenia being one of the most researched conditions in this regard. In individuals with schizophrenia, the most prominent saccadic abnormalities are inhibition errors and decreased spatial accuracy during volitional saccades (Broerse et al., 2001). However, it is essential to note that neither deficits are exclusive to individuals with schizophrenia. Our experimental results show promise for developing a non-invasive risk stratification tool, as we could accurately distinguish MCI patients from CN controls using EM behavior assessed during PS/AS and Go/No-go tasks.

The study has limitations, as EM changes can occur with several neurological conditions and MCI subtypes (e.g., amnestic vs. nonamnestic conditions) (Garbutt et al., 2008; Molitor et al., 2015). Eye-tracking can be used in multiple neurological disorders, but more research with diverse samples is needed to test this possibility. Additionally, eye-tracking depends on good visual function in subjects and requires high-performance devices, which can limit its availability despite minimal staff training needs.

In summary, during the trials, patients with MCI had difficulty maintaining fixation, suppressing saccades, or making saccades toward or away from the target. The results show that EM metrics may reveal impaired sustained attention, working memory, and executive control function in patients with MCI performing EM tasks. This study shows that machine learning can aid in automatically detecting cognitive impairment using eye-tracking data. The classification model with EM metrics performed better than the model with demographic characteristics, indicating the usefulness of EM feature analysis for early-stage cognitive decline detection. Combining EM metrics, demographic characteristics, and MMSE scores resulted in the best classification performance. Combining these modalities improves model performance and demonstrates that EM metrics, demographic characteristics, and MMSE data are complementary in detecting cognitive impairment. Our study, which had a sample size of approximately 600 participants, the largest sample size of a clinical trial in this area to date, provides strong evidence for the effectiveness of EM metrics in screening patients with MCI and has significant implications for clinical practice.

The following are our primary contributions: first, we built a dataset that includes EM data collected during interleaved EM tasks, brief cognitive test scores, and demographic information. Then, we used this dataset to study the contribution of the EM data to the classification of patients with MCI vs. CN controls, showing that EM data collected noninvasively could discriminate between patients with MCI and CN controls. Finally, we showed that EM data collected during an interleaved EM task complemented demographic and MMSE score data in detecting subtle cognitive impairment.

## Data availability statement

The raw data supporting the conclusions of this article will be made available by the authors, without undue reservation.

## Ethics statement

The studies involving human participants were reviewed and approved by Chonnam National University Hospital Institutional Review Board (IRB no CNUH-2019-279). The patients/participants provided their written informed consent to participate in this study.

## Author contributions

JO, BK, JIK, and JUK conceived the manuscript. Data verification was carried out by JO and JIK. JO and BK performed the statistical analyses and generated the figures. Drafting of the manuscript was done by JO, BK, JIK, and JUK. Methodology and administration of the trial were overseen by JIK, KHL, and JUK. All authors contributed to the article and approved the submitted version.

## Funding

The Korean Government funded this study under a grant (KSN1823130) to the Korea Institute of Oriental Medicine.

## Conflict of interest

The authors declare that the research was conducted in the absence of any commercial or financial relationships that could be construed as a potential conflict of interest.

## Publisher’s note

All claims expressed in this article are solely those of the authors and do not necessarily represent those of their affiliated organizations, or those of the publisher, the editors and the reviewers. Any product that may be evaluated in this article, or claim that may be made by its manufacturer, is not guaranteed or endorsed by the publisher.
