# Erratum: Eye movement changes as an indicator of mild cognitive impairment

**DOI:** 10.3389/fnins.2023.1243766

**Published:** 2023-07-13

**Authors:** Frontiers Production Office

**Affiliations:** Frontiers Media SA, Lausanne, Switzerland

**Keywords:** Alzheimer's disease, mild cognitive impairment, eye movement analysis and synthesis, machine learning (ML), saccades

Due to a production error, there was a mistake in Figures 1, 2 as published. These two Figures were not included in the main text instead Supplementary Figures A1 and A2 were included. The corrected Figures 1, 2 appear below.

**Figure 1:**
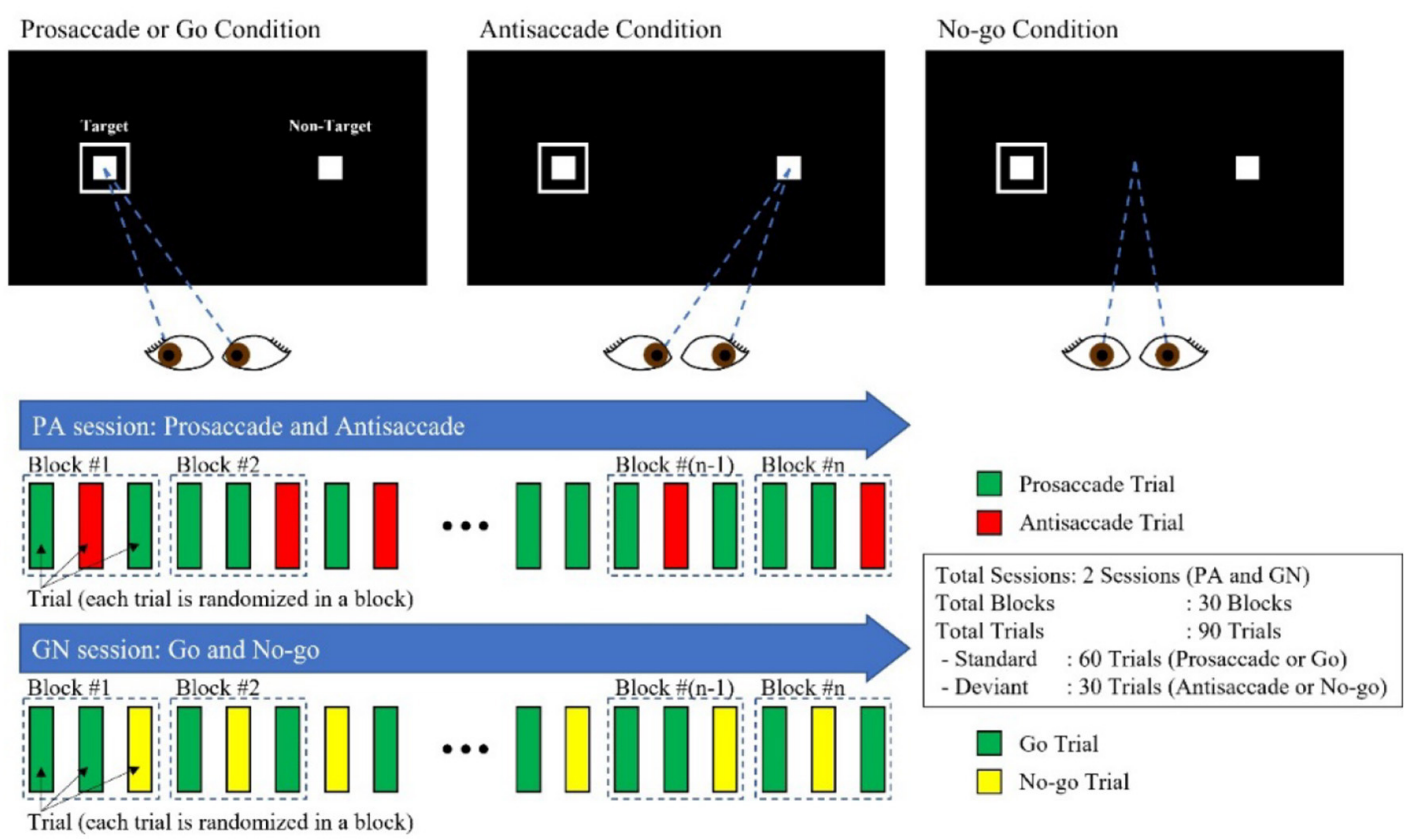
PS/AS and Go/No-go paradigms.

**Figure 2:**
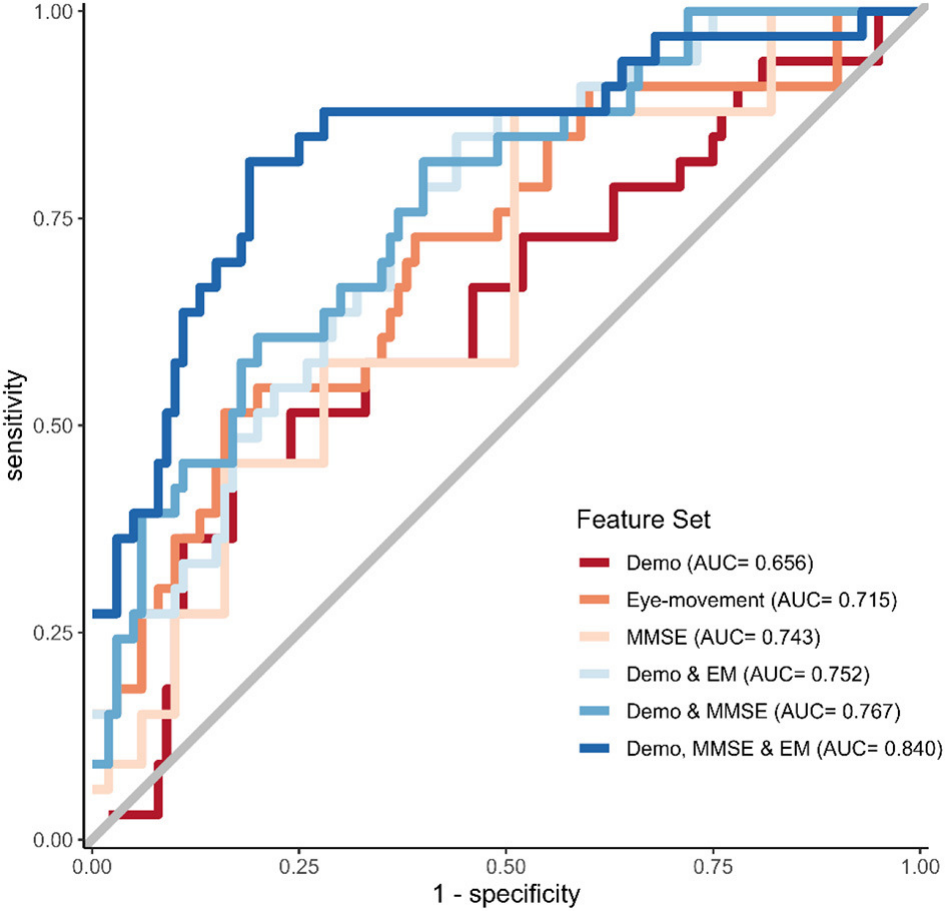
ROC curves for the best-performing prediction models per feature set.

Due to a production error, there was a mistake in the **Supplementary material** as published. The incorrect Data Sheet was published, omitting Figures A1 and A2 The correct Data Sheet can now be found under the original articles link: https://www.frontiersin.org/articles/10.3389/fnins.2023.1171417/full#supplementary-material

The publisher apologizes for this mistake. The original article has been updated.

