# Supplementary material for: Eye movement changes as an indicator of mild cognitive impairment
Source: Front Neurosci. 2023 Jun 15;17:1171417. doi: 10.3389/fnins.2023.1171417 (PMC10307957; doi:10.3389/fnins.2023.1171417)

**Appendix**

| **Table A1: Sensitivity and Specificity results of prediction models according to feature set and classification model** | | | | | | | | | | | |
| --- | --- | --- | --- | --- | --- | --- | --- | --- | --- | --- | --- |
|  | Logistic Regression | |  | Random Forest | |  | Support Vector Machine | |  | Extreme Gradient Boosting | |
|  | Sens | Spec |  | Sens | Spec |  | Sens | Spec |  | Sens | Spec |
| **Demo** | 0.541 | 0.624 |  | 0.482 | 0.694 |  | 0.667 | 0.455 |  | 0.576 | 0.515 |
| **MMSE** | 0.847 | 0.729 |  | 0.847 | 0.906 |  | 0.455 | 0.576 |  | 0.455 | 0.273 |
| **EM** | 0.647 | 0.847 |  | 0.671 | 0.718 |  | 0.636 | 0.455 |  | 0.515 | 0.545 |
| **Demo + MMSE** | 0.765 | 0.788 |  | 0.729 | 0.718 |  | 0.606 | 0.364 |  | 0.636 | 0.515 |
| **Demo + EM** | 0.647 | 0.871 |  | 0.659 | 0.682 |  | 0.697 | 0.333 |  | 0.697 | 0.636 |
| **Demo + MMSE +EM** | 0.635 | 0.906 |  | 0.624 | 0.753 |  | 0.697 | 0.545 |  | 0.727 | 0.818 |
| LR: Logistic Regression, RF: Random Forest, Sens: Sensitivity, Spec: Specificity, SVM: Support Vector Machine, XGB: Gradient-boost trees. | | | | | | | | | | | |

**(D)**

**(C)**

**(B)**

**(A)**

**Figure. A1.** Correlation of PS/AS responses and demographics (age and level of education) and MMSE for (A) PS responses by NC (B) PS responses by MCI patients (C) AS responses by NC (D) AS responses by MCI patients. The correlation coefficients arranged by hierarchical clustering, displaying only those correlations that were found to be statistically significant. Non-significant correlations are represented by blanks.


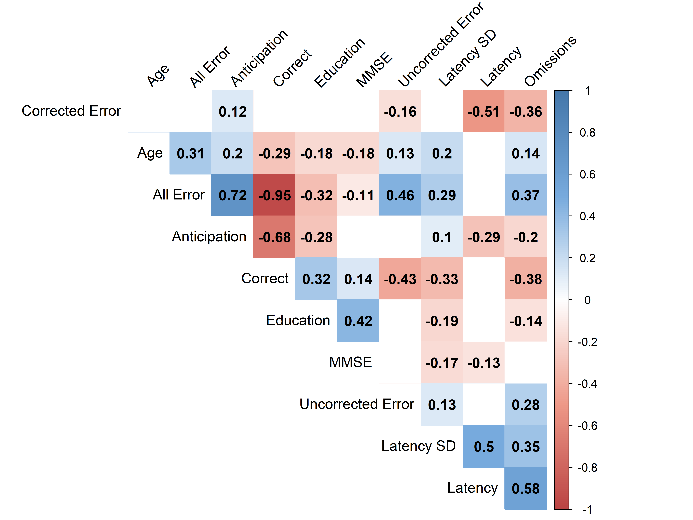

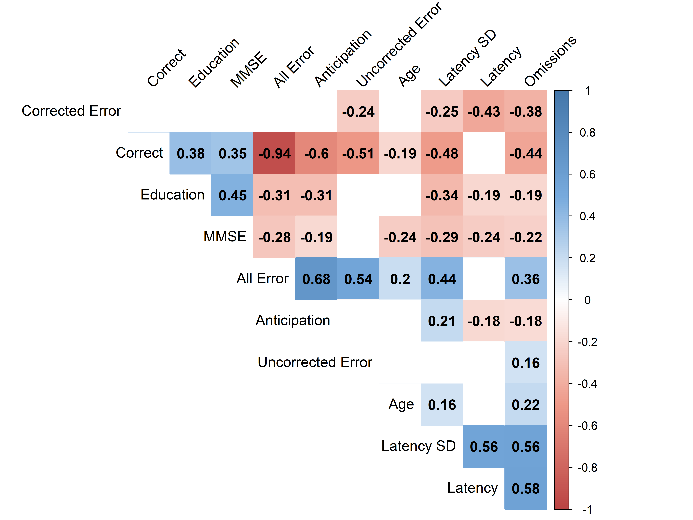

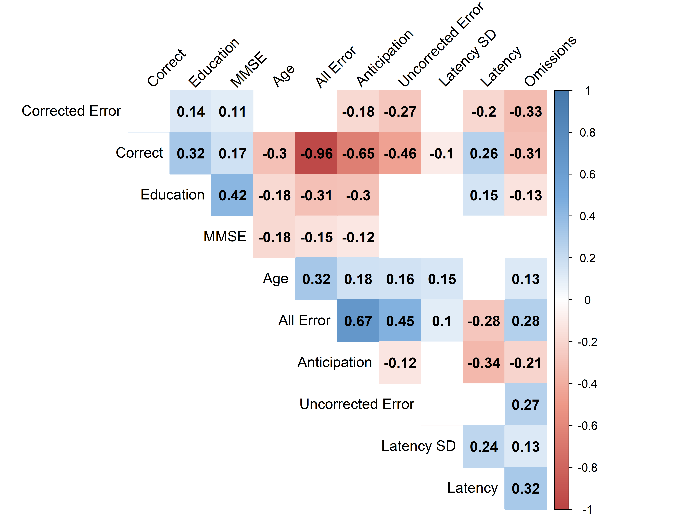

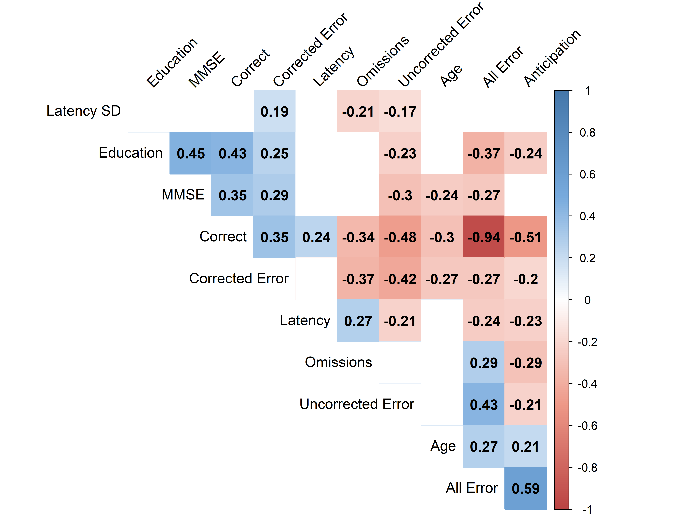


**(D)**

**(C)**

**(B)**

**(A)**

**Figure. A2.** Correlation of GO/NG responses and demographics (age and level of education) and MMSE for (A) GO responses by NC (B) GO responses by MCI patients (C) NG responses by NC (D) NG responses by MCI patients. The correlation coefficients arranged by hierarchical clustering, displaying only those correlations that were found to be statistically significant. Non-significant correlations are represented by blanks.


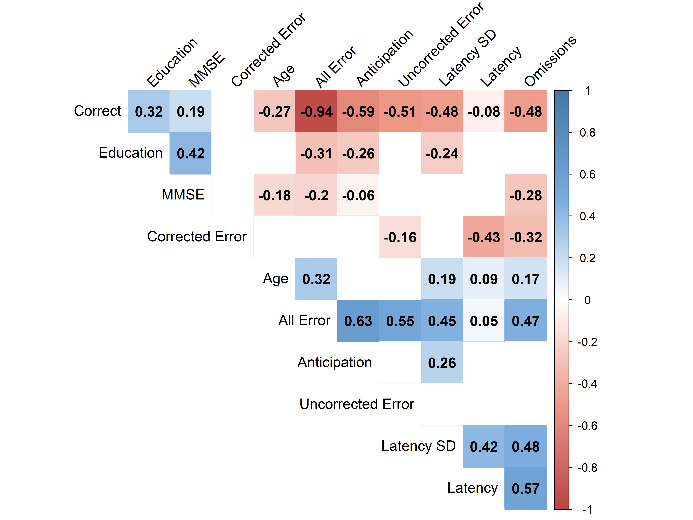

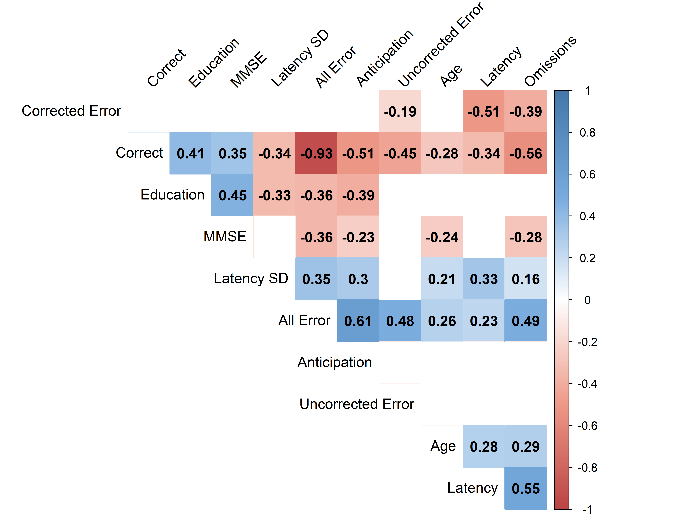

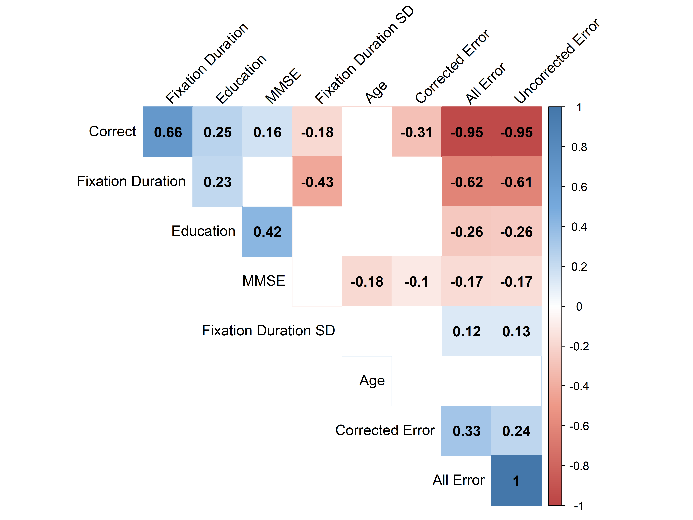

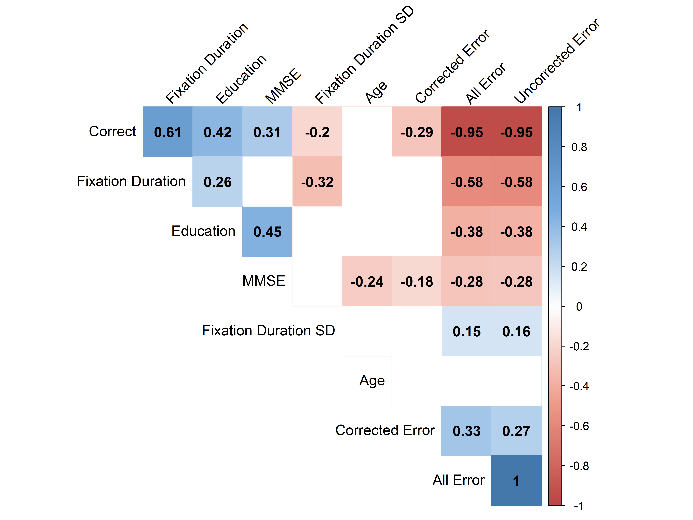

Supplement: Supplementary file 1 [file Data_Sheet_1.docx]
